# The extraction of complex relationships and their conversion to biological expression language (BEL) overview of the BioCreative VI (2017) BEL track

**DOI:** 10.1093/database/baz084

**Published:** 2019-10-11

**Authors:** Sumit Madan, Justyna Szostak, Ravikumar Komandur Elayavilli, Richard Tzong-Han Tsai, Mehdi Ali, Longhua Qian, Majid Rastegar-Mojarad, Julia Hoeng, Juliane Fluck

**Affiliations:** 1 Fraunhofer Institute for Algorithms and Scientific Computing, Schloss Birlinghoven, 53754 Sankt Augustin, Germany; 2 Philip Morris International R&D, Philip Morris Products S.A., Quai Jeanrenaud 5, 2000 Neuchatel, Switzerland; 3 Department of Health Sciences Research, Mayo Clinic, 200 First St. SW, Rochester, MN 55905, USA; 4 Department of Computer Science and Information Engineering, National Central University, Taiwan, R.O.C., Taiwan 320; 5 NLP Lab, School of Computer Science and Technology, Soochow University, Suzhou, 215006 Suzhou, China; 6 Friedrich Wilhelm University of Bonn, 53012 Bonn, Germany

## Abstract

Knowledge of the molecular interactions of biological and chemical entities and their involvement in biological processes or clinical phenotypes is important for data interpretation. Unfortunately, this knowledge is mostly embedded in the literature in such a way that it is unavailable for automated data analysis procedures. Biological expression language (BEL) is a syntax representation allowing for the structured representation of a broad range of biological relationships. It is used in various situations to extract such knowledge and transform it into BEL networks. To support the tedious and time-intensive extraction work of curators with automated methods, we developed the BEL track within the framework of BioCreative Challenges. Within the BEL track, we provide training data and an evaluation environment to encourage the text mining community to tackle the automatic extraction of complex BEL relationships. In 2017 BioCreative VI, the 2015 BEL track was repeated with new test data. Although only minor improvements in text snippet retrieval for given statements were achieved during this second BEL task iteration, a significant increase of BEL statement extraction performance from provided sentences could be seen. The best performing system reached a 32% F-score for the extraction of complete BEL statements and with the given named entities this increased to 49%. This time, besides rule-based systems, new methods involving hierarchical sequence labeling and neural networks were applied for BEL statement extraction.

## Introduction

Identifying the molecular mechanisms influencing disease development and therapeutic responses is currently one of the most promising ways of identifying better treatments for non-responders to traditional treatments. There is an increasing amount of research with large-scale biological data sets produced to identify those mechanisms, and the results are mainly published in the scientific literature. To use this data for further analysis, it needs to be in a computer-readable format. To achieve this, availability of a syntax and the conversion of the biological data and knowledge to this syntax are required. For dynamic models, systems biology markup language (SBML) is the main format (1) used, and for traditional pathway information, the exchange format BioPAX (2) is useful. Both formats contain representations of biological mechanisms or models rather than findings from literature. Also, most relationships described in the literature are difficult to convert directly to these representations. Furthermore, they are not easily accessible to humans without a user interface. As an alternative, biological expression language (BEL) has been developed to allow the representation of the huge variety of causal relationships expressed in the literature (3). BEL is better suited for biocuration to encode findings or observations from the literature compared to other representation formats. Additionally, BEL follows the principles of FAIR (findable, accessible, interoperable and reusable) data, providing the necessary metadata to make it findable and enabling interoperability using standardized terminologies and external data sources for all biological entities. Using further metadata, BEL also allows for the fine granular annotation of data. Additionally, it is well suited to integration into knowledge graphs or graphical network analyses (4).

However, it is labor-intensive to extract the relevant information from the primary literature and convert the free text data into structured relationships using controlled vocabularies (5). In recent years, research has been conducted to enable the automatic extraction of biological relationships and their translation into BEL. First, a syntax for BEL conversion was developed from the text mining-focused representation of relationships provided by Biomedical Natural Language Processing shared tasks (6). These tasks provide fined grained and linguistically motivated annotations for biologically relevant extractions (http://2016.bionlp-st.org/). Second, to enable a broader text mining community to extract relationships in BEL format, BEL was introduced within BioCreative 2015 as a new track (7, 8). For this track, a subset of relationships containing genes, proteins, chemical compounds, biological processes and diseases was used (9). In the first BioCreative BEL track, the best performing system reached an F-score of 22% for the extraction of complete BEL statements (8). Despite the low F-score for complete relationships, core relationships containing relationship partners were extracted with an F-score of up to 49% (8). These results support the possibility of the semi-automatic extraction of relationships, supporting curation experts with the facility of automated extraction. BEL information extraction workflow (BELIEF) supports such semi-automatic curation (10). BELIEF (http://belief.scai.fraunhofer.de/BeliefDashboard) is a public web service with underlying text mining workflows and a curation interface that enables the semi-automatic extraction of complex relationships, and their coding, in BEL.

In 2017, the BEL track was conducted a second time in the context of BioCreative VI. Experiences in other areas have demonstrated that repetitions of tasks tend to result in increased performance. For the 2017 BEL track, novel, and yet unpublished, test data from the disease ulcerative colitis (11, 12) was created. The BioCreative 2015 test set and 2015 annotated result set are available for the participating groups (https://wiki.openbel.org/display/BIOC/Datasets). Here, the track details, relevant resources and newly created test set are described. Furthermore, the participating systems present their solutions and performance results from the new BEL track.

## BEL track overview

### BEL and used namespaces

**Figure 1:**
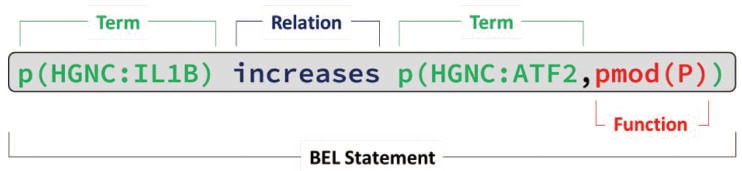
Example of a BEL statement extracted from the sentence ‘IL-1β caused a time-dependent increase in Caco-2 ATF-2 phosphorylation, starting at 10 min of treatment (Fig. 3A).’ (46) (PMID: 23656735) (BEL:201720027; identifier in BEL corpus). The BEL statement consists of two protein abundances [*p (HGNC:IL1B)* and *p (HGNC:ATF2)*], one protein modification function representing a phosphorylation event [*pmod(P)*] and a relationship type (*increases*).

BEL statements encode semantic triples with subject, relationship and object. An example BEL statement and its corresponding sentence are shown in Figure 1. For the BEL track, we focused on two causal relationship types: increase and decrease. Using normalized entities from so-called namespaces for subject and objects, the resulting statements can be integrated and merged into networks as well as aligned to other data. These namespaces are generated from database entries {e.g. human genes from HGNC [HGNC stands for HUGO Gene Nomenclature Committee (http://www.genenames.org)], mouse genes from MGI [MGI stands for Mouse Genome Informatics (http://www.informatics.jax.org/)] and chemical entities from the ChEBI [ChEBI stands for Chemical Entities of Biological Interest (https://www.ebi.ac.uk/chebi/)] database}. In our example in Figure 1, textual references such as IL1-ß and ATF-2 are normalized to the HGNC database entries IL1B and ATF2, respectively. The reference to the chosen namespace is given using a prefix with the database short name separated by a colon, ‘HGNC’ in our example. Other namespaces originate either from ontologies, such as the biological processes subtree (GO:0008150) of the Gene Ontology for GOBP (GOBP stands for Gene Ontology Biological Process) or from terminologies, such as the diseases namespace MESHD (MESH disease subtree is available at https://meshb.nlm.nih.gov/treeView.) from the Medical Subject Headings diseases subtree.

For the different entities, different class abundances are assigned: the abundance function *a()* is assigned to chemicals, *bp()* for biological processes and *path()* (pathology) for diseases. For genes, different abundances *g()* (gene), *r()* (mRNA) or *p()* (protein) are possible, but to reduce complexity only *p()* is used in the BEL track. In our example in Figure 1, we used the protein abundance function for both entities. In addition to class abundances, different functions can also be assigned to the biological entities. For the BEL track, we focused on the protein phosphorylation function *pmod(P)*, the protein activity function *act()*, the translocation function *tloc()*, the protein degradation function *deg()* and finally the *complex()* function, to describe protein complexes. For a more detailed description of this, see Rinaldi *et al.* (8). In Figure 1, the syntax for describing a phosphorylated protein is: *p (HGNC:ATF2, pmod(P))*.

## Task description and evaluation

The BEL track challenge was organized into two tasks evaluating the complementary aspects of the problem:

(i) Task 1: given textual evidence for a BEL statement, generate the corresponding BEL statement.
(ii) Task 2: given a BEL statement, provide a maximum of 10 additional evidence sentences.

The extraction of relationships and their coding in BEL is a complex task as a multitude of entity, relationship and function types can be involved in a single relationship. Therefore, we made a number of simplifications for the evaluation. Briefly, for genes and proteins, HGNC, EntrezGene or mouse orthologous (MGI) identifiers were accepted. For the abundance functions of these namespaces, all correct abundances were accepted. Furthermore, for the modification function *pmod()* and the translocation function *tloc()*, the number of arguments was reduced. The details of all simplifications are provided online (http://wiki.openbel.org/display/BIOC/All+Functions+Evaluation+Overview).

A cascade evaluation model with different levels of success rates was performed for syntactically valid statements, whereas invalid statements were ignored. A submitted full BEL statement was automatically cut into its fragments to enable evaluations on lower levels. On the term level, only the correctness of BEL terms was assessed. Furthermore, the correctness of the discovered entities, the associated namespaces and the associated abundance and process functions were measured.

The correctness of the discovered function was evaluated on the function level. Functions were only accepted together with their argument, the BEL term. As a simplification, a complex function was only valid if at least one of its arguments was correct. On the secondary function level, the correctness of a function alone was measured, regardless of the correctness of its term-arguments.

**Figure 2:**
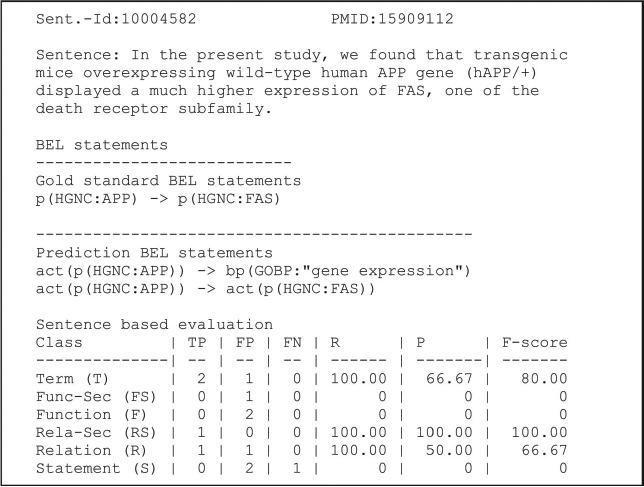
An example of a candidate evaluation. The example shows the candidate sentence, the gold standard and predicted statements. The scores are provided for all primary and secondary levels (8). Abbreviations: PMID (PubMed identifier), true positive (TP), false positive (FP), false negative (FN), recall (R), precision (P). Adapted and reprinted with permission from Fluck *et al.* (7).

In the relationship level evaluation, only the terms and relationships were considered. Functions that were part of a BEL statement were not taken into account on this level. Yet again, two levels of evaluation were considered. To obtain a full score relationship, subject, object and the relationship type had to be correct. For the secondary relationship level, partial relationships containing two out of three correct units (subject, object and relationship type) were considered fulfilled. This level was introduced in order to give weighting to the results, which although were partially correct, could still be useful as suggestions to a human curator in the context of a semi-automated approach. Finally, we evaluated how many BEL statements were entirely correct. The cascade evaluation model is depicted in Figure 2 and described in detail by Rinaldi *et al.* (8).

For task 2, up to 10 evidence sentences for each BEL statement were accepted from the participating systems. Those statements were evaluated on two levels: on the ‘fully supportive level’, the sentence had to contain all necessary information for a biologist to create the BEL statement; and on the ‘partially supportive level’, the sentence was correct when relevant context information from surrounding sentences contained the additional information required. For more detailed information on the evaluation criteria, see Rinaldi *et al.* (8).

## Materials and methods

### Training data and preparation of new test set

The training data and test data from BioCreative V (2015) are available online (https://wiki.openbel.org/display/BIOC/Datasets). The description of the training set selection and curation are described in detail by Fluck *et al.* (9). For the generation of the 2017 BEL track task 1 test set, we used a real-world case and extracted new data in the disease context of ulcerative colitis. For the test set, we restricted the named entity classes to those that could be normalized to the gene and protein namespaces HGNC and MGI, including ChEBI for chemical names, MESHD for disease names and GOBP for biological processes. We also used a restricted set of relationship types and functions as defined above. For the extraction, we used automatic support in the form of BELIEF (10). This workflow pre-annotated and normalized the named entities and suggested BEL statements in a user-friendly curation environment. At the front-end, the curator could browse through the text, search for unrecognized named entities and edit or add statements. Only user-selected statements were exported by the system.

In the first step, two curators independently extracted the information from the same full texts. In the comparison of results, it became clear that it is very tedious to extract all possible statements from a document. Especially, since we would extract repetitively very similar statements only with different experimental settings, which was irrelevant for task 1. It was also not feasible for independent curators to select the same sentences for curation in the full text. Therefore, we decided to take a more straightforward approach. One person selected the relevant sentences and extracted all of the BEL statements from this sentence. Then, the second curator analyzed and edited this set. Finally, differences were discussed in an annotation jamboree.

An overview of the number of different entities, functions and relationship types of the task 1 test set is given in Table 1. For task 2, BEL statements were curated from PubMed abstracts to make sure that at least one sentence could be found for every BEL statement.

**Table 1:** Distribution of term, function and relationship types in the training and test corpora

**Type**	**Training**	**Test 2015**	**Test 2017**
***Terms***			
p()	19.918	346	328
a()	1.927	37	52
bp()	877	31	23
path()	244	15	2
***Functions***			
act()	6.332	36	79
pmod()	1.411	9	36
complex()	750	15	5
tloc()	406	13	10
deg()	205	6	4
sub()	23	0	0
trunc()	6	0	0
***Relationships***			
increases	8.112	155	130
decreases	2.956	53	68

For task 1 stage 1, we also provided the normalized names of all biological processes occurring in the test set, as extracting such concepts remains a non-trivial task. Finally, for task 1 stage 2, we provided a file with entity information and offsets and the associated normalized concept with the namespace.

### Supporting material

The participants were provided with a range of supporting resources and comprehensive documentation (https://wiki.openbel.org/display/BIOC/BioCreative+BEL+Task+Challenges), containing a description of the formats and a detailed explanation of the evaluation process. The evaluation of the different levels of a single BEL statement was illustrated using a set of concrete example submissions as reference. Additionally, validation and evaluation interfaces (http://bio-eval.scai.fraunhofer.de/cgi-bin/General_server.rc ) were provided for the participants to validate and test their generated statements during the development phase. The BEL statement validator checks the user-provided BEL statements with respect to formal correctness and provides specific error messages for invalid BEL statements. For the sample, training and 2015 test sets, the evaluation interface evaluated the input BEL statements based on evaluation criteria such as term, function, relationship and full statement level. For a more detailed description of the candidate evaluation, see Rinaldi *et al.* (8).

Resources for further support included BEL statements from the training, sample and 2015 test sets in BioC format. A tab-separated format that contained all fragments of the BEL statements (terms, functions and relations) was automatically generated from the sample, training and 2015 test sets. These were provided to the participants as supporting material [c.f. (8)] (https://wiki.openbel.org/display/BIOC/Datasets).

### Participating systems

All participating systems can be compartmentalized in at least two main parts—a first part that is composed of a number of named entity recognition (NER) components for the recognition and normalization of the entities and a second part for relationship extraction and BEL translation. In NER, the systems use different tools that utilize various algorithmic approaches such as dictionary, rule-based and machine learning. The second part is dedicated to relationship extraction. Here, three approaches use a dependency parser as a preprocess step. BELMiner 2.0 uses graph traversal and BelSmile extracts relationships using predicate argument structures. After the extraction, all systems have a final component to transform extracted relationships to BEL statements.

Two systems, the graph-based traversal system BELMiner 2.0 and the semantic role labeling approach BelSmile, participated in the BEL track for the second time. The newly participating systems used either hierarchical sequence labeling or deep neural networks for relationship extraction. Finally, one system, BelTracker, participated in task 2 of the BEL track, using a pre-annotated corpus for the different named entities and integrating a ranking component to order the resulting sentences by relevance. All systems are described in the following sections.

#### BELMiner 2.0—information extraction system to extract BEL relationships from biomedical literature

BELMiner 2.0 (13) is a generic graph-based traversal on the dependency graphs constructed from entity-normalized sentences. The following components are executed in sequence to process the evidence statement, with each component incrementally contributing towards BEL statement extraction: (i) extraction of normalized entities, (ii) identification of dependency structure, (iii) graph-based traversal to extract causal relationships, (iv) formalization of causal relations into BEL statements and (v) filtering out of irrelevant BEL statements.

**Figure 3:**
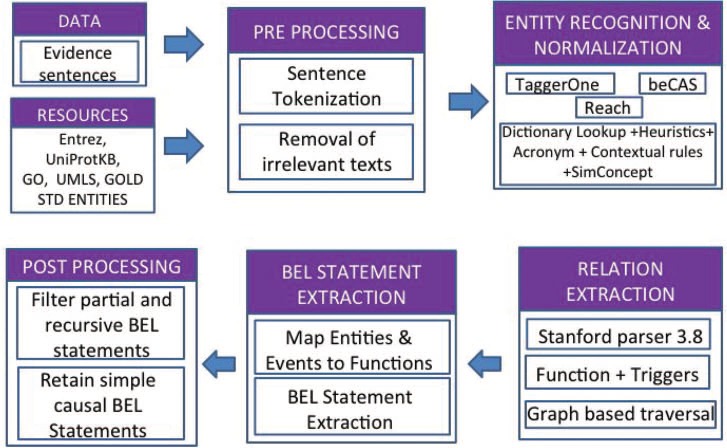
BELMiner 2.0 architecture.

Figure 3 outlines the overall architecture of BELMiner 2.0. It consists of an ensemble of state-of-the-art entity normalization tools to extract normalized entities from the evidence sentences provided for each NER task. TaggerOne (14), a machine-learning toolkit, was adapted for BEL entity recognition and supplemented with annotations from other external tools such as beCAS (15) and Reach (16).

Within BelMiner2.0, the Stanford Parser 3.8 (17) is applied to identify extended dependencies such as anaphora and co-references, and appositives and dependencies that occur beyond clausal boundaries. The identification of appropriate arguments for functions and relations involves a graph-based traversal (18). The system first identifies the event or activity and phrase or term in the sentence and, using that as an anchor, traverses bi-directionally along the dependency graph to identify the arguments. During graph traversal, the system considers certain properties of the node, such as the semantic type of the node and properties such as negations. It also identifies the successive double negation of events along the traversal path to correctly identify the type of main event.

#### BelSmile—a semantic role labeling approach for extracting BEL statements

**Figure 4:**
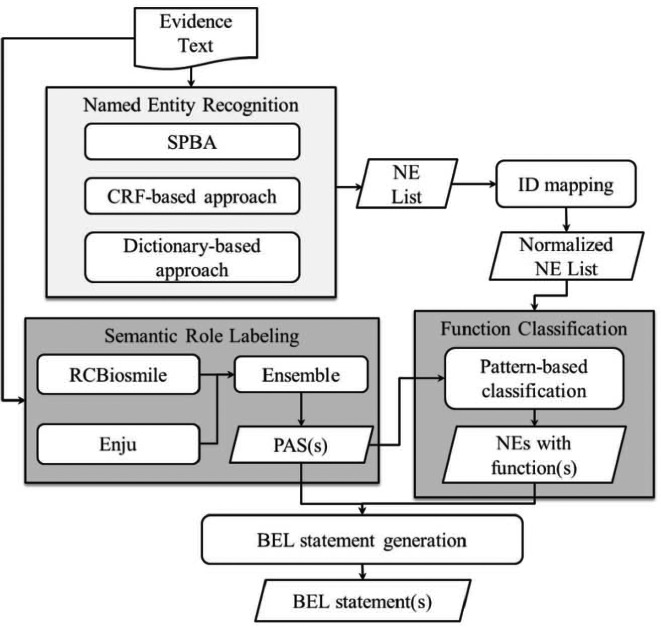
BelSmile workflow.

BelSmile (19) contains two main natural language processing (NLP) stages: NER and semantic role labeling (SRL). Figure 4 shows the workflow of the system. In the NER stage, BelSmile consists of an ensemble system composed of three approaches: statistical principle, conditional random fields (CRF) and dictionary-based. The statistical principle-based approach is used to identify protein mentions and achieved the highest score in terms of the second evaluation metric of the BioCreative V.5 Gene and protein related object recognition (GPRO) task (20). The CRF-based NERChem (21) is used to identify chemical mentions. Finally, the dictionary-based approach is used to recognize disease and biological process mentions by using external dictionaries including Entrez, ChEBI and BEL official dictionaries, which are also used to normalize each recognized NE mention to its database identifier.

In the SRL stage, two systems, RCBiosmile (22) and Enju (23), are employed. Enju covers some predicates (verbs) not recognized by RCBiosmile, which is trained on the BioProp corpus (24). In the configuration achieving the highest score, predicate argument structures (PASs) extracted from both systems are used.

After the two NLP stages, BelSmile’s nominal patterns (25) and newly-compiled verbal patterns are used to generate the BEL function markup for each named entity mention. These verbal patterns refer to elements in a PAS. Finally, BelSmile generates BEL-level statement(s) by feeding the PASs and named entities with functions into the BelSmile’s statement generation module.

#### A hierarchical sequence labeling system for the BioCreative VI BEL task

**Figure 5:**
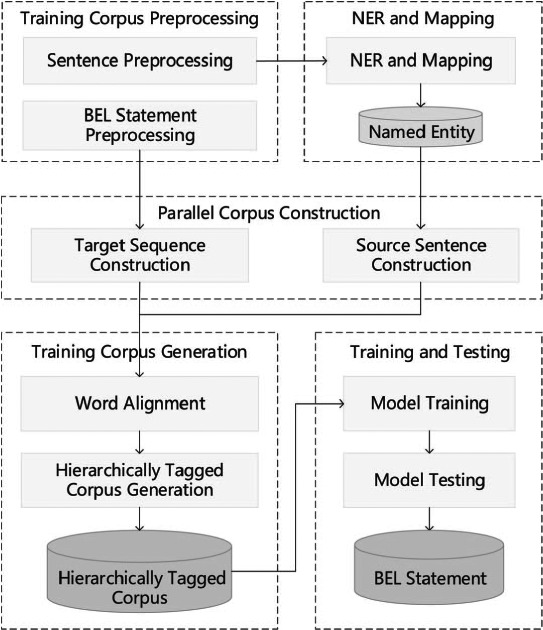
Hierarchical sequence labeling system pipeline.

The hierarchical sequence labeling system (26) pipeline consists of five components: preprocessing, NER and mapping, parallel corpus construction, training corpus generation and model training and testing. Figure 5 illustrates the framework of the system.

*Preprocessing:* Preprocessing the training corpus includes two steps: rule-based sentence tokenization and BEL statement serialization. The latter step also reduces BEL statement redundancy and inconsistencies and elevates the hierarchical level of some protein modification functions.

*NER and mapping:* Three NER tools are used to identify biomedical entities, including GNormplus (27), tmChem (28) and DNorm (29). Additionally, a dictionary-based search method is employed to promote the recall rate of entities. For the training corpus, the identified entities are mapped to those in BEL statements.

**Figure 6:**
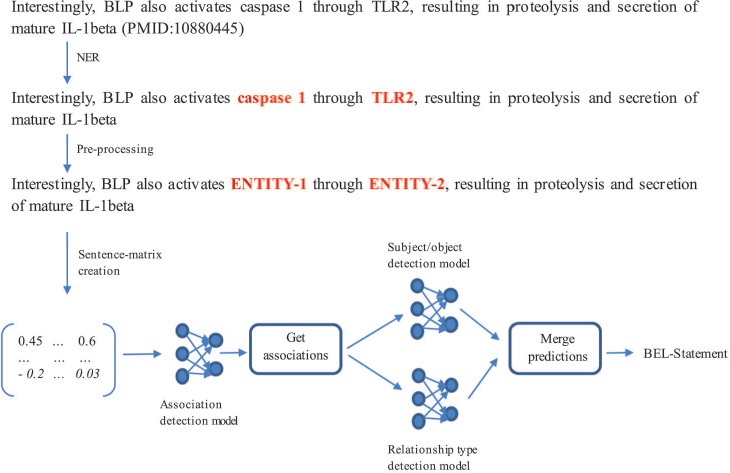
Architecture of the neural network-based system.

*Parallel corpus construction:* In order to obtain the alignments between entities, functions and relationships in the BEL statement and the words in the sentence, we recast this problem as the word alignment problem between the source language (text sentence) and the target language (serial representation of the BEL statement). The target language is generated through three stages: (i) BEL tree generation, (ii) BEL tree unification and (iii) BEL tree serialization. To obtain the source language, the sentences are tokenized and then simplified by extracting the minimal subtree containing all the entities in the BEL statement. In a further step, words are serialized in the subtree according to their original order.

*Training corpus generation:* Generating a training corpus from the aforementioned parallel corpus follows two steps: initially, a word alignment tool GIZA++ (30) is utilized to obtain alignments between words and BEL nodes. A hierarchically tagged corpus is then generated based on the alignment results between the nodes in the BEL statement and the words in the sentence. The corpus is annotated using the ‘BIESO’ (B-beginning, I-intermediate, E-end, S-single entity, O-outside) labeling scheme.

*Training and testing:* The open source CRF package CRF++ (31) is used to train hierarchical sequence labeling models from the hierarchically tagged corpus. The first level sequence labeling model is trained on words and entities. When training the k-th level model, the lower k-1 layers are treated as features and the top-level model is reached recursively.

In the testing stage, the trained *L* models are employed to label the test examples. In contrast to during training, when labeling the k-th layer the labels automatically recognized in the lower k-1 layers are treated as features. After labeling all the layers, the labeling results are converted into BEL statements. This process is basically the reverse of training example generation and can be divided into three steps, that is, BEL tree generation, unified tree splitting and BEL statement generation.

#### A neural network-based system to extract BEL statements

BEL statement extraction through the neural network-based system (32) is divided into four subtasks (c.f. Figure 6): (i) NER, (ii) association detection, (iii) subject and object detection and (iv) relationship type detection. In the following, we discuss how we solved each subtask and present the network architecture that we used.

*NER:* For NER, the rule- and dictionary-based software ProMiner (33) was used. It contains several terminologies to detect named entities. For the training set, we used ProMiner to find the offsets of the annotated entities. For the test set, we considered all detected entities for further predictions.

*Association detection model:* The second subtask was to decide whether a sentence describes an association between two entities or not (independently from any information about subject and object and the relationship type). In order to create artificial negative examples, a pair of entities was annotated as a negative example when no relationship was annotated in the training set. This strategy can produce false positives as shown in (9) but can be realized with very low effort. The model was trained based on 6389 instances comprising 4633 positive and 1756 negative examples.

*Subject and object detection model:* The third subtask was to assign the subject and object within an entity pair. If the subject appears before the object, the instance is assigned to the class ‘Subject First’, otherwise it is assigned to the class ‘Object First’. This training set consists of 4633 examples from which 3156 instances belong to the class ‘Subject First’ and 1477 instances to the class ‘Object First’.

*Relationship type detection model*: The last subtask was to determine the type of relationship of an entity pair participating in an association. After mapping the relationship types ‘directly increases’ to ‘increases’ and ‘directly decreases’ to ‘decreases’, a neural network for this task was trained based on 4325 instances consisting of 3103 ‘increases’ and 1222 ‘decreases’ examples.

*Architecture of the multichannel convolutional neural networks (CNN):* For all three models described, B-D multichannel CNNs were trained (32). The system development is based on the work of Quan *et al.* (34) and Hua *et al.* (35). The embedding layer contains the representations of the input sentence. The multichannel CNN uses different input channels for different representations of the sentence. Four Word2Vec models trained by Pyysalo *et al.* (36), which are based on PubMed, PubMed Central and Wikipedia, are used to transform each word of the sentence into a vector representation. Based on these word vectors, a sentence-matrix is generated and passed to the network as input. In the sentence-matrix, each column represents a word, and the number of rows indicates the dimension of each word vector.

In the convolutional layer, local features are computed by applying a convolutional operation on each sentence representation (32). For each sentence representation, we retrieved a scalar value every time the sliding window of the convolution operation shifted. The scalar values of each shift were summed and passed to the activation function. The rows of a new matrix represent the results of the convolutions created by different kernels/filters (in this case four filters). At the end of the convolutional layer, a max-pooling operation created a feature vector by extracting the most significant features, taking the biggest value for each row. The feature vector was passed to a fully connected layer and the result given as input to a softmax classifier producing the predictions. In the convolutional layer and in the fully connected layer, the exponential linear unit was used as the non-linear activation function.

#### BELTracker—a system for semantic sentence retrieval

The BELTracker (37) is focused on the second task of the BEL track, in which evidence sentences for given BEL statements are requested. The system has three main components: indexing, retrieval and ranking. In the first component, we retrieved ‘informative sentences’ from MEDLINE abstracts and indexed them in a text search engine, called Elasticsearch (https://www.elastic.co/de/products/elasticsearch ). To identify such sentences, the system relied on the Semantic Medline Database (SemMedDB) (38), which is a relational database that stores all sentences from MEDLINE abstracts containing at least two biomedical entities and a relationship between them.

The retrieval component in this system was the same as in our previous system (39). All elements (e.g. entities, functions and relationships) in the given BEL statement were identified, synonyms were added and a query for the index was generated. Elasticsearch retrieves these sentences based on co-occurrence returns and, at most, 1000 evidence sentences for a given BEL statement.

In order to rank the retrieved sentences, the ranking component applies three classifiers: entity–entity (EE) classifier, function–entity (FE) classifier and relationship classifier. The EE classifier examines the relationship between the entities and classifies sentences into ‘positive’ (existence of a relationship) and ‘negative’ (no relation; only co-occurrence). To obtain negative instances, sentences from SemMedDB that contain two biomedical entities with a ‘co-exist’ relationship type (meaning there is not any specific type of relationship between the entities and they only co-occur in corresponding sentences) were retrieved. Unigrams, bigrams and word embedding of terms between entities were used as features for the EE classifier.

The ‘function–entity classifier’ calculated the probability of a relationship between functions and entities in the retrieved sentences. For example, the BEL statement ‘catalyticActivity (HGNC:XIAP) decreases catalyticActivity (HGNC:CASP9)’ contains two FE relationships: ‘cat-XIAP’ and ‘cat-CASP9’. We trained a set of FE classifiers, where each classifier targeted one BEL function. To train FE classifiers, both positive and negative instances were available in the training data provided by the organizers. Unigrams and bigrams of the words surrounding an entity (a window of three–five words) were utilized as features for FE classifiers. Beside lexical features, we used word embedding, dependency-based word embedding and abstract meaning representation embedding (40) as other features for FE classifiers.

**Figure 7:**
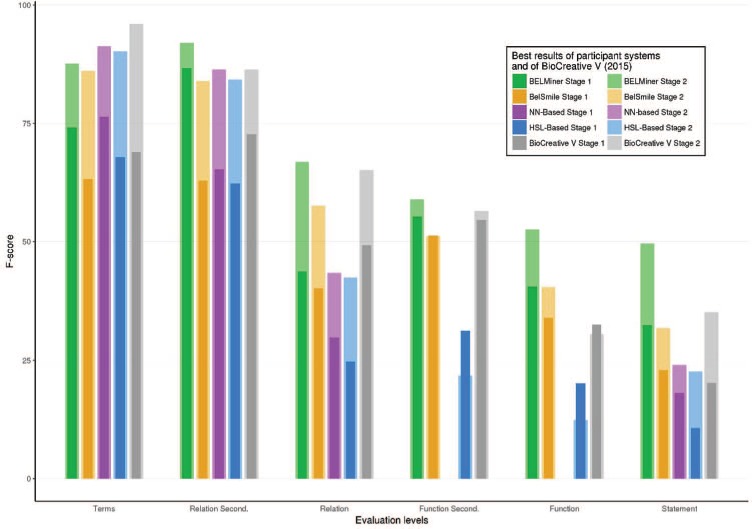
Best results of each system of BioCreative VI (2017) and BioCreative V (2015) in each structured level of task 1.

The last classifier categorized the retrieved sentences based on two BEL relationship types: ‘increase’ and ‘decrease’. More details about this classifier, such as list of the features, are explained in our previous publication (39).

The final score was calculated using the following equation:

Score_sentence_ = W_EE_ * P_EE_ + W_FE_ * P_FE_ + W_R_ * P_R_.

*P* represents the probability produced by the classifiers and *W* indicates the weight assigned to each classifier. We assigned weight to each classifier based on its importance and the accuracy of the training data set. The weights were: W_EE_ = 0.4, W_FE_ = 0.5 and W_Relation_ = 0.1. The FE classifier had the highest weight because the data used to train this classifier had less noise compared with the EE classifier. The relation classifier had a lower weight compared with the others, because we observed that rarely did two sentences contain all elements of a BEL statement, but they did often convey two different relationship types. That is, if entities and functions of a given BEL statement appear in a sentence, it is likely that the sentence has the relationship type mentioned in the BEL statement.

## Results

Here, we describe the achieved performance scores of the participating systems for each BEL track task.

### Task 1: given textual evidence for a BEL statement, generate the corresponding BEL statement

Thirteen teams registered for the first task of the BEL track; however, four teams contributed results of their BEL extraction systems. The task was performed in two stages. In the first stage, teams had to provide their own NER and normalization to the defined namespaces, whereas in the second stage, the entities of the relationships were given. In each stage, a maximum number of three submissions were permitted. As described in the methods section, the evaluation of the submitted BEL statements was performed in a number of subsiding steps. This allowed for the identification of the challenges on each different structural level.

A summary of the best F-scores for all participants of stage 1 is shown in Figure 7, along with the best scores of the BioCreative V (2015) evaluation. Although different test sets were used in the BioCreative V (2015) and VI (2017) BEL tracks, their characteristics, such as the size of the datasets, the included entity types, BEL functions and the relationships, were similar. It is therefore reasonable to compare the results of both assessments. Overall, Figure 7 illustrates several trends: firstly, prediction scores of the secondary level are significantly higher than their specific primary level scores; secondly, the complexity of the extraction process increases from term, relation and function to BEL statement extraction level. Compared with BioCreative V, a clear performance gain was achieved at the functional and full statement levels.

The detailed results of each individual run of stage 1 are provided in Table 2. The results are color-coded according to F-score values (F), the main evaluation criterion, and supplemented by the values for precision (P) and recall (R). The best results for each evaluation metric are marked in bold. In general, all teams took part on all structural levels except the neural network-based system, which excluded the function level.

**Table 2:** Evaluation of stage 1 of task 1 (prediction of BEL statements without gold standard entities). F, P and R stand for F-score, precision and recall, respectively

**System**		**Terms**			**Function**			**Function-Second**			**Relation**			**Relation-Second**			**Statement**		
	**Run**	**F**	**P**	**R**	**F**	**P**	**R**	**F**	**P**	**R**	**F**	**P**	**R**	**F**	**P**	**R**	**F**	**P**	**R**
**BEL-**	r1	63.24	84.62	50.49	33.99	44.83	27.37	51.24	67.39	41.33	40.22	55.38	31.58	62.92	88.19	48.91	22.99	33.33	17.54
**smile**	r2	57.75	81.93	44.59	31.08	43.4	24.21	38.67	69.05	38.67	36.78	53.33	28.07	57.73	86.84	43.23	20.71	31.82	15.35
	r3	61.24	88.27	46.89	32.88	47.06	25.26	46.15	64.29	36	37.43	56.14	28.07	62.03	92.24	46.72	21.15	33.98	15.35
**HSL-**	r1	50.88	76.82	38.03	6	60	3.16	7.5	60	4	16.77	31.71	11.4	45.14	80	31.44	7.38	15.71	4.82
**based**	r2	55.29	81.01	41.97	6.06	75	3.16	7.59	75	4	21.52	38.64	14.91	51.06	84	36.68	10.67	22.22	7.02
	r3	67.83	72.22	63.93	20.17	50	12.63	31.25	71.43	20	24.69	28.25	21.93	62.25	70.95	55.46	10.44	12.9	8.77
**BEL-**	r1	74.14	78.18	70.49	**40.54**	56.6	31.58	**55.28**	70.83	45.33	43.65	51.81	37.72	86.17	89.62	82.97	32.28	40.67	26.75
**miner**	**r2**	72.89	78.71	67.87	40.29	63.64	29.47	54.39	79.49	41.33	**43.77**	52.12	37.72	**86.71**	93	81.22	**32.45**	41.22	26.75
**NN-**	r1	**76.39**	81.18	72.13	0	0	0	0	0	0	29.87	25.55	35.96	65.19	60.45	70.74	18.08	16.1	20.61
**based**	r2	**76.39**	81.18	72.13	0	0	0	0	0	0	28.92	24.19	35.96	65.23	59.29	72.49	17.88	15.53	21.05

Overall, the two systems participating the second time performed best in almost all categories: the BELMiner system, in particular, outperformed all other systems. Analysis of the different steps revealed that NER performance in stage 1 reached F-scores between 63% and 76.39% for the best performing runs. The function extraction seemed to be more difficult than relationship extraction and produced the greatest differences. BELMiner could extract function information in stage 1 with an F-score of 40.54% and BelSmile with a best performance of 33.99%. The hierarchical sequence labeling system could recognize correct functions with an F-score of only 20%, and the neural network-based system did not predict any functions at all.

At the relationship level, BELMiner and BelSmile achieved similar F-score performances of 43.77% and 40.22%, respectively. This is a large difference with the other systems with F-scores of 29.8% and 24.6% for the neural network-based system and the hierarchical sequence labeling system, respectively. At the secondary relationship level, where only two thirds of the relationship (subject, object and relation type) needed to be correct, BELMiner achieved an impressive F-score of 87%. All other systems reached F-scores only between 62% and 65%.

Finally, the best full BEL statement extraction score for BELMiner was 32.45% at stage 1. This illustrates the difficulty of this highly structured prediction task. Despite similar values for relationship extraction, BelSmile lost performance in comparison with BELMiner and achieved an F-score of only 22.99%. Surprisingly, despite no function recognition and low relationship extraction performance, the neural network-based system obtained an F-score for full statement extraction of 18%. The hierarchical sequence labeling system reached only 10.6% and shows the difficulty of sentence to BEL statement alignment.

The detailed results for task 1 stage 2 are shown in Table 3, and a summary is illustrated in Figure 7. In this stage, the gold standard concepts, together with their specific text spans, were made available to the teams. All teams could significantly benefit and improve on the level of the full statements. These results show the importance of high-quality term recognition for further higher-level recognition tasks. NER was enhanced to 83–91% in the second stage. Because NER was not evaluated independently, but only when full statements were provided by the systems, no system could reach an F-score of 100%.

**Table 3:** Evaluation of stage 2 of task 1 (prediction of BEL statements with gold standard entities)

**System**		**Terms**			**Function**			**Function-second**			**Relation**			**Relation-second**			**Statement**		
	**Run**	**F**	**P**	**R**	**F**	**P**	**R**	**F**	**P**	**R**	**F**	**P**	**R**	**F**	**P**	**R**	**F**	**P**	**R**
**BEL-**	r1	83.93	99.11	72.79	36.36	47.46	29.47	46.77	59.18	38.67	57.22	73.29	46.93	83.33	98.8	72.05	31.30	46.15	23.68
**smile**	r2	86.09	99.15	76.07	40.51	50.79	33.68	51.16	61.11	44	56.08	70.67	46.49	83.92	98.82	72.93	30.95	44.63	23.68
	r3	85.45	99.13	75.08	39.24	49.21	32.63	50	60.38	42.67	57.6	73.47	47.37	83.63	98.81	72.49	31.79	46.61	24.12
**HSL-** **based**	r1	90.20	98.83	82.95	12.39	38.89	7.37	21.74	58.82	13.33	42.52	52.94	35.53	84.24	96.61	74.67	22.66	32	17.54
**BEL-**	r1	87.65	90.56	84.92	51.75	77.08	38.95	57.63	79.07	45.33	**66.83**	76.7	59.21	**92.06**	95.75	88.65	49.2	63.01	40.35
**miner**	**r2**	86.4	90.94	82.3	**52.55**	85.71	37.89	**58.93**	89.19	44	**66.83**	76.7	59.21	91.92	97.55	86.9	**49.6**	64.34	40.35
	r3	87.41	90.81	84.26	51.75	77.08	38.95	57.63	79.07	45.33	**66.83**	76.7	59.21	91.99	96.63	87.77	49.2	63.01	40.35
**NN-**	r1	**91.33**	99.23	84.59	0	0	0	0	0	0	43.51	41.6	45.61	86.36	90.05	82.97	23.61	25	22.37
**based**	r2	88.36	99.18	79.67	0	0	0	0	0	0	42.47	44.29	40.79	83.41	91.19	76.86	24.06	28.07	21.05
	r3	76.71	98.96	62.62	0	0	0	0	0	0	25.68	29.38	22.81	71.76	85.98	61.57	15.06	18.47	12.72

Performance increases can be seen on all evaluation levels for all teams. Again, BELMiner improved the most on all levels. For full statement level, it reached the highest F-score of 49.6% with the provided terms. In comparison to stage 1, the score was increased up to 17% on the same test set. Similar performance increases were seen on the function and relationship levels too. In summary, the comparison of all teams shows that most teams, except for the neural network-based system, built systems that focused more on precision rather than recall. Furthermore, high scores on the relationship level do not necessarily correlate with high scores on the full statement level. This is because the full statement level combines all structural levels.

**Figure 8:**
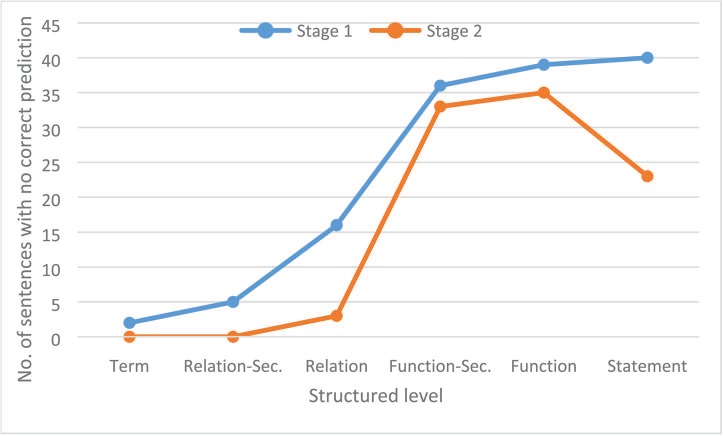
Number of sentences for each structured level on each stage for which no correct prediction was produced by any run of any participant system.

To evaluate whether some information is more difficult to extract than others, we identified the information for which not a single correct prediction was produced. Figure 8 shows the number of sentences for which no run of any participant system provided any valid prediction for each structured level. On the term level, only two sentences with no true term predictions were found. In stage 1, on the relationship-secondary and relationship level, no correct predictions were made for 6 and 15 sentences, respectively. In stage 2, where the terms were provided, this was reduced significantly. A high number of sentences with no valid predictions for function-secondary and function level in both stages could be identified (36 and 39 in stage 1, 33 and 35 in stage 2). Here, the given entity information in stage 2 only slightly reduced the amount of non-prediction sentences. The analysis of the different functions showed that around 23% (18/79) of the activity functions and 50% of the *tloc()* (5/10) functions were not predicted from any system. For the activity functions, *tloc()* is also the most ambiguous in curator annotation and is also most difficult to recognize because biological interpretation is necessary. An example is the statement ‘p (HGNC:TNF) increases tloc(p (HGNC:KHDRBS1), GOCC:cytoplasm, GOCC:nucleus)’ that was extracted from the following sentence: ‘The results showed Sam68 levels decreased in the cytoplasm following TNF-a stimulation for 1 h, which was paralleled by a corresponding increase in its nuclear level showing that Sam68 is a TNF-a responsive protein (Fig. 7d)’ (41).

Figure 8 also clearly shows that entity information also improved performance on the statement level. In stage 1, 40 sentences lacked a correct prediction, whereas in stage 2, only 23 sentences had no valid prediction. A typical relationship that the automatic systems could not extract was the BEL statement ‘act(p (HGNC:EDNRA)) increases a (CHEBI:“calcium(2+)”)’ from the sentence ‘This ET-1-induced cell proliferation and [Ca2+] increase were completely abolished by BQ123, a selective ETAR antagonist, but not by BQ788, a selective ETBR antagonist’ (42). For the extraction of such statements, an understanding of biological experiments and reasoning, as well as interpretation of their results, is necessary. For the example provided, it can be reasoned that activity is necessary for the read-out if the removal of a protein activity [in this case *act(p (HGNC:EDNRA))* through its antagonist *BQ123*] leads to the abolishment of the read-out (in this case an increase of *[Ca2+]*).

### Task 2: given a BEL statement, provide a maximum of 10 additional evidence sentences

Only one system, BELTracker, participated in this task. In agreement with the organizers, two runs with two different configurations and only five ranked sentences for each run were submitted. The correctness of the provided evidence sentences was evaluated manually and rated on two different levels of strictness:

*(i) Fully supportive*: Relationship is fully expressed in the sentence.

*(ii) Partially supportive*: Relationship can be extracted from the sentence if context sentences or biological background knowledge are taken into account.

To evaluate the quality of the curation results, we calculated an inter-annotator agreement. For this task, part of the manual curation was performed by two different curators. For 150 entries, we observed a high agreement of 93% (kappa statistic: 0.75) and 91% (kappa statistic: 0.79) for the categories fully and partially supportive, respectively.

As shown in Table 4 the system provided 382 evidence sentences for 98 BEL statements in each run (mean 3.9 sentences per statement). In run 1, for 55 BEL statements, there was at least one entirely correct evidence sentence; for 71 statements, there was at least one sentence meeting the partially supportive evaluation condition; and in run 2, 58 and 70 BEL statements satisfied the fully and partially supportive evaluation conditions, respectively.

**Table 4:** Evaluation results of task 2 including MAP

**Runs**	**Criterion**	**TP**	**FP**	**Precision**	**MAP**
Run 1	Full	117	265	30.6%	59.6%
	Partial	175	207	45.8%	77.5%
Run 2	Full	121	261	31.7%	50.2%
	Partial	192	190	50.3%	76.7%

Table 4 also shows the detailed numbers for true positives (TP), false positives (FP) and the resulting precision at the micro level. Around one-third of all sentences fully expressed the desired relationship. To assess the ranking quality of the system, we computed the mean average precision (MAP). Although the first run had a slightly lower precision compared with the second run, the MAP was considerably higher, especially for full supportive sentences. Overall, based on the results and the low number of participants, task 2 appeared to be as difficult as task 1. For some statements, only a few references could be extracted by curators and no prediction was provided by the automatic system. One example is the statement ‘p (MGI:Tmsb4x) decreases path (MESHD:Colitis)’. Only the abstract with the PMID, *28127198,* could be found containing these entities and also this relation. A second example is the statement ‘p (HGNC:CXCL8) increases p (HGNC:BCL2)’*,* where numerous abstracts could be found with those co-occurring genes but not the relationship. For this example, BELTracker made only false predictions.

Placeholder for Table 4.

## Discussion

The results of biomedical analyses are complex and are often associated with several experimental settings including inhibitors, silencing or knock-out experiments. For understanding and interpreting these results, and encoding the findings as relationships, biological background knowledge is often necessary. Biologists encoding such relationships most likely select evidence sentences on the basis of the detailed experimental information included. All this leads to a high complexity for relationship extraction. Furthermore, results are reported not only on a molecular level but often on biological process or disease levels as well. The BEL corpora created for the BioCreative VI track 4 added a new resource for use in the training and evaluation of biological relationship extraction methods. In contrast with other published corpora, BEL includes multimodal relationships spanning from molecular protein–protein or protein–chemical entity relationships to higher-level causal relationships including biological processes or diseases, which are common in real-world data, to tackle real-world use cases. There are already a number of publicly available corpora addressing different relationship types (43–45). In contrast with the BEL corpora, they mostly address only one or two types of entity.

Consequently, the BEL track remains the most complex task within the BioCreative challenge, even after our attempts to simplify the BEL statements and the evaluation. In this track, four different entity types, gene and protein names, small chemicals, disease names and biological processes have to be recognized and normalized for the term-level evaluation. The performance of term detection ranges between F-scores of 50.88% and 76.39%. This scale of NER performance dramatically influences the results of subsequent higher levels. By providing the term information in the second stage of task 1, the NER performance within submitted BEL statements increased to F-score values between 83.93% and 91.33%. Hence, all other extraction steps for function, relationship type and full statement extraction improved drastically because of the provided name entities. For example, the best system, BELMiner 2.0, improved the BEL statement extraction performance from an F-score of 32.45% to an F-score of 49.6%. Primary researchers could support information extraction tremendously in using standardized terminology in their publications.

The second most influential factor was the past experience of the team that utilized the systems created for the past 2015 BEL track. BelSmile and BELMiner 2.0 participated again this second time. Furthermore, both systems rely on dependency parsed text as well as rules applied on dependencies. Rules can be more easily learned on smaller training sets and do not need in depth annotation of the training data. In direct comparison, BELMiner 2.0, using graph-based traversal, outperformed BelSmile, which is based on SRL for relation extraction. Performance differences for these systems can be seen on all levels: term recognition, function recognition and, to a lesser extent, relationship extraction. The other newly introduced methods are more dependent on positional information and the size of the training set. The system based on hierarchical sequence labeling aligned the BEL statements and the sentence to generate the corpus. Also, the neural network-based system relies on positional information, as do most currently available machine learning algorithms. Both approaches would most likely gain performance with exhaustive positional annotations and larger training sets.

The biological research community needs highly precise data for their analyses to further understand biological mechanisms. Therefore, most of the biological databases that provide research and experimental data focus mainly on manual curation for relationship extraction to meet the quality needs of the research community. The comparison of the secondary and primary levels of task 1 shows that the precision and recall values of secondary levels are high enough to use as recommendations for manual curation. Therefore, we believe that migrating from manual to semi-automatic curation workflows, by using the BEL extraction systems mentioned here, is worth consideration to still produce high quality curation data while at the same time raising curation efficiency and reducing time effort.

Another important aspect of scientific research is the reproducibility of findings. In general, reproducibility increases the confidence in findings, and is reused by other researchers for their own studies. Finding reproduced evidence from the scientific literature is a challenging text mining problem. Task 2 tried to tackle this challenge by providing a platform for building systems that can find further evidence for a given BEL-encoded relationship. The evaluation of task 2 was split into two levels: fully and partially supportive. The only system participating in this task performed with around 30% precision for finding up to five instances of evidence for a single relationship. As the size of databases is rising enormously, we think this will become more important to the research community in the future.

Most current and popular methods used by NLP system developers and applicants are based on machine learning. For those methods, larger training sets and positional annotation information needs to be available. Therefore, for future track development for BEL extraction, we plan to focus on three different areas. Firstly, we would like to further reduce the complexity of the task and focus on certain subtypes of entities and relationships. Secondly, we will attempt to increase the size of training data and, in particular, provide further positional annotations. Also, the data produced by previous BioCreative challenges might represent a useful resource that can be utilized in the future as training data for the BEL track. Finally, we need to provide tools that reduce the time needed for participants to build complex BEL-related workflows so that they can focus more on the mining tasks.

A mixture of automatic and manual post-processing steps is still necessary for the generation of high-quality data. Better alignment of curators with unambiguous annotation guidelines and more interdisciplinary work of both text miners and curators are necessary to overcome some of this additional work. This will hopefully lead to better training corpora, as well as methods supporting efficient curation in the future.

## Conclusion

The extraction of molecular mechanism information with a fully automatic relationship extraction is a complex task. Through the second round of the BEL track and the provision of training data, the performance of participating systems increased drastically. When entity annotation was given, an F-score of up to 49% could be reached. Nevertheless, NER is the most relevant information extraction task to accomplish such an F-score. An increase in further training data and terminology resources is necessary to improve performance. Additionally, to enhance participation in such a complex track, we have to consider subdividing the task further, find automatic ways to increase the amount of training data and provide more supportive tools for each individual subtask. Nevertheless, we are positive that the investment (task definitions, data sets, documentation, evaluation framework and participating systems) made so far in the BEL track shows the need for BEL-related text mining in the scientific community.

## Notes

*Availability*: The BEL track resources are available at https://wiki.openbel.org/display/BIOC/BioCreative+BEL+Task+Challenges. The participating systems are not publicly accessible.

**Database URL**: https://wiki.openbel.org/display/BIOC/BioCreative+BEL+Task+Challenges
